# Comparative proteomic analysis reveals different responses in porcine lymph nodes to virulent and attenuated homologous African swine fever virus strains

**DOI:** 10.1186/s13567-018-0585-z

**Published:** 2018-09-12

**Authors:** Júber Herrera-Uribe, Ángeles Jiménez-Marín, Anna Lacasta, Paula L. Monteagudo, Sonia Pina-Pedrero, Fernando Rodríguez, Ángela Moreno, Juan J. Garrido

**Affiliations:** 1 Grupo de Genómica y Mejora Animal, Departamento de Genética, Facultad de Veterinaria, Universidad de Córdoba, Córdoba, Spain; 2 International Livestock Research Intitute (ILRI), Nairobi, 00100 Kenya; 3 Centre de Recerca En Sanitat Animal (CReSA), Institut de Recerca i Tecnologia Agroalimentàries (IRTA), Campus UAB, Bellaterra, 08193 Barcelona, Spain; 4 Instituto de Agricultura Sostenible, Campus Alameda del Obispo, 14080 CSIC Córdoba, Spain

## Abstract

African swine fever (ASF) is a pathology of pigs against which there is no treatment or vaccine. Understanding the equilibrium between innate and adaptive protective responses and immune pathology might contribute to the development of strategies against ASFV. Here we compare, using a proteomic approach, the course of the in vivo infection caused by two homologous strains: the virulent E75 and the attenuated E75CV1. Our results show a progressive loss of proteins by day 7 post-infection (pi) with E75, reflecting tissue destruction. Many signal pathways were affected by both infections but in different ways and extensions. Cytoskeletal remodelling and clathrin-endocytosis were affected by both isolates, while a greater number of proteins involved on inflammatory and immunological pathways were altered by E75CV1. 14-3-3 mediated signalling, related to immunity and apoptosis, was inhibited by both isolates. The implication of the Rho GTPases by E75CV1 throughout infection is also evident. Early events reflected the lack of E75 recognition by the immune system, an evasion strategy acquired by the virulent strains, and significant changes at 7 days post-infection (dpi), coinciding with the peak of infection and the time of death. The protein signature at day 31 pi with E75CV1 seems to reflect events observed at 1 dpi, including the upregulation of proteosomal subunits and molecules described as autoantigens (vimentin, HSPB1, enolase and lymphocyte cytosolic protein 1), which allow the speculation that auto-antibodies could contribute to chronic ASFV infections. Therefore, the use of proteomics could help understand ASFV pathogenesis and immune protection, opening new avenues for future research.

**Electronic supplementary material:**

The online version of this article (10.1186/s13567-018-0585-z) contains supplementary material, which is available to authorized users.

## Introduction

African swine fever virus (ASFV) is the causal agent of a haemorrhagic and often-lethal porcine disease, African swine fever (ASF), which causes affected countries important economic losses. There is no vaccine available against the disease, albeit promising developments for future implementation are being currently developed [1]. ASF may range from an acute, highly lethal infection to subclinical chronic forms, depending on a complex contribution of viral and host factors [2]. The pig immune response to ASFV has been widely studied [3, 4], showing that the virus has effective mechanisms of evading pig defensive systems, thus contributing to the immune pathology observed during acute ASF, and to virus persistence in its hosts [5]. Studies about virus-cells interaction have contributed significantly to unravel the mechanisms involved in pig response [3, 6–10]. In this regard, it has been shown that the ASFV genome encodes a large number of genes that have been identified as playing a role in host immune evasion including: interferon (IFN) inhibition by several multigene family members [11], the NF-κB and NFAT inhibitor A238L or the apoptosis inhibitor A179L, among others. All these aspects have recently been reviewed [12]. In addition, it is known that ASFV controls host transcription and cellular machinery of protein synthesis [13] thus contributing to pathology. The high complexity of ASFV, together with its tropism for the immune system of the host, complicates the understanding of ASF pathogenesis. Both ASFV-specific antibodies [4] and CD8^+^ T-cells [3, 14], have been postulated as key players in the protection against ASFV. The dual role of the immune system during ASFV infection becomes evident again during chronic ASF-infections, characterized by mild clinical signs associated, on occasions, with immunopathological processes, such as immune complexes and swine IgM, IgG and C1q depositions [15]. Swine macrophages are the main target for ASFV, and depending on multiple factors, including the virulence of the ASFV strain, the immune system can play a dual role during both, ASFV-protection but also contributing to the virus pathogenesis. As mentioned above, virulent ASFV strains can evade the early recognition of the immune system, strategies that facilitate their replication and in vivo dissemination that, in the last stages of acute infection, provoke tissue destruction, leukopenia and total dysregulation of the immune system, reflected with the typical storm of cytokines thus contributing to ASF acute pathogenesis [16]. Conversely, infections with attenuated ASFV strains normally render subclinical infections that are rapidly recognized by the innate immune system and cleared from the body, yielding pigs capable of resisting homologous lethal challenge [16].

Here we extend these studies by presenting a comparative proteomic analysis using gastrohepatic lymph nodes (GLN) from pigs infected in vivo with either the attenuated E75CV1 strain or with E75, its parental virulent ASFV strain, at different days post-infection (dpi); a model system previously used to unmask some of the mechanisms involved in homologous protection against ASFV [16]. Since ASFV-infection modifies the expression patterns of host cell proteins, the application of proteomic approaches might help to clarify the intrinsic mechanism involved in ASFV-host interactions, as has been demonstrated [10, 17]. These methodologies have contributed to better understandings of how some viruses reprogram cell resources for their own benefit, or avoid host defensive mechanisms to survive in their host [18], but a limited number of proteomic studies have been carried out with swine pathogens. Thus, proteomic strategies have been used to study the differences observed after in vitro infection of Vero cells with virulent or attenuated strains of porcine epidemic diarrhoea virus [19], or PK-15 cells infected with classical swine fever virus [20]. Additionally, our group has previously described the protein profile of lymph nodes of piglets inoculated with Porcine circovirus type 2 (PCV2) [21]. Two proteomic studies have been published so far for ASFV infection: the first one focussed on proteins differentially expressed after ASFV-macrophage infection, although they were not specifically identified [17] and the second one, using Vero cells infected with the 608VR13 ASFV isolate, identifying proteins involved in apoptosis or in the transcription modulation [10].

In this work, we have conducted the first 2-DE proteomic approach and bioinformatic data analysis using lymph node tissue from pigs infected with two homologous ASFV strains with distinct virulence: the tissue culture adapted E75CV1, an attenuated virus, and its parental virulent E75 virus. In order to reach a deeper understanding of pig-ASFV interactions and swine immune responses, samples were obtained at different times post-infection. This large- scale proteomic study should provide a complete view of the major and important functions and pathways altered during the infection process, and hopefully, it could be used in the future to develop anti-ASFV strategies.

## Materials and methods

### Experimental design for the in vivo experiment

The in vivo experiment has been previously described and the animals and samples used in this analysis are the same as those used in experiment 2 of our previous study [16]. Briefly, a group of 24 pigs was infected with 104 HAU50 of E75CV1, a second group of 18 pigs was infected using the same dose and route of the virulent E75 and finally a third group of 12 pigs remained uninfected (control group). Pigs from the three groups were sacrificed at days 1, 3 and 7 pi (6 or 3 per group and day) and 6 pigs from the attenuated E75CV1 and 3 from the control group were also sacrificed at day 31 pi. Lymph node samples of all animals (54 pigs) were analysed by 2-DE and mass spectrometry (see below).

### Two-dimensional gel electrophoresis (2-DE) and image analysis

GLN were subjected to mechanical dissociation in sample buffer (7 M urea, 2 M thiourea, 4% w/v CHAPS, 1% w/v DTT, 0.8% ampholytes, 0.2 M PMSF) by scraping and gentle squeezing. Each supernatant was recovered and, after clean up precipitation, lymph node protein extracts for each condition (control, E75-infected or E75CV1-infected) and day (1, 3, 7 and 31 dpi) were pooled and analyzed by 2-DE following previously published methods [22]. Briefly, protein extracts were diluted in rehydration buffer (7 M urea, 2 M thiourea, 4% w/v CHAPS, 20 mM DTT, 0.5% Triton X-100, 0.5% ampholytes) and immobilized pH gradient strips of 17 cm (5–8 linear pH gradient, Bio-Rad) were rehydrated with 300 μL (500 μg) of each protein solution and focused in a PROTEAN IEF Cell (Bio-Rad) using the following parameters: (1) active rehydration at 50 V for 12 h; (2) at 250 V for 15 min without pause after rehydratation; (3) rapid ramp until reaching 10 000 V/h and (4) until 80 000 V/h with slow ramped voltage. Previously, protein profile was analyzed by 2-DE on 3–10 pH gradient strips, finding that the resolved spots were concentrated in the 5–8 pH range. Second dimension was performed on 12% SDS–polyacrylamide gels using PROTEAN PlusTM Dodeca (Bio-Rad). Four replicated for each of the conditions (virulent, attenuated or control) were analyzed simultaneously for each dpi.

Gels were stained with SYPRO Ruby protein gel stain (Bio-Rad). Gel images were digitized with the FX Pro Plus Multi imager system (Bio-Rad) and analyzed with the PD Quest version 7.3 software (Bio-Rad). Spots detected by the program were matched between each gel in each group. Normalized protein spot volume (area multiplied by stain intensity) was calculated for each spot in the control sample and compared to its counterpart (sample from ASFV-infected animals). Intensity data were used to calculate differences in protein expression between groups (controls vs. E75 or E75CV1 infected samples), for each dpi, using a Student’s *t* test (*p* < 0.05) (two-tailed with unequal variances) after checking normality by the Wilks–Shapiro test. Differentially expressed spots were selected for protein identification by mass spectrometry.

### Protein identification by mass spectrometry

Spots were automatically excised in a ProPic station (Genomic Solutions) and digested with modified porcine trypsin (sequencing grade; Promega), by using a ProGest digestion station (Genomic Solutions), as previously described [22]. Briefly, gel pieces were destained with ammonium bicarbonate/acetonitrile (ACN), and subsequently subjected to dehydration/rehydration cycles and dried. Gel pieces were digested with trypsin, peptides extracted with trichloroacetic acid and desalted and concentrated by using μC-18 ZipTip columns (Millipore) in a ProMS station (Genomic Solutions). After that were loaded onto a 4800 MALDI-TOF/TOF Analyzer (Applied Biosystems) in automatic mode with the following setting: for the Mass Spectrometry (MS) data, m/z range 800–4000 with an accelerating voltage of 20 kV, peak density of maximum 50 peaks per 200 Da, minimal S/N ratio of 10 and maximum peak at 65. Spectra were internally calibrated with peptides from trypsin autolysis (M + H+= 842.509, M + H+= 2211.104). For the MS/MS data, fragment selection criteria were a minimum signal/noise (S/N) ratio of 5, a maximum number of peaks set at 65 and peak density of maximum 50 peaks per 200 Da. For each precursor selected for MS/MS analysis, fragment mass values in the range from 60 to 10 Da below precursor mass were used to peptide identification. Protein identification was assigned by peptide mass fingerprinting and confirmed by MS/MS analysis of at least three peptides in each sample. Mascot 1.9 search engine (Matrixscience) was used for protein identification running on GPS software (Applied Biosystems) against the NCBI mammalian database (updated monthly). Only those proteins with a significant protein score (*p* < 0.05) according to Mascot were taken into account in subsequent analysis.

### Functional analysis of the proteins

Differentially regulated proteins were analyzed through the use of Ingenuity Pathway Analysis software (IPA, Ingenuity^®^ Systems). This system identifies the functions and canonical pathways that are most significant to the data set, supported by canonical information stored in the Ingenuity Knowledge Base. Fisher’s exact test was used to calculate a *p* value determining the probability that the association between the proteins in the dataset and the functions and canonical pathways is explained by chance alone. These *p*-values are calculated based on the number of proteins that participate in a given function or pathway relative to the total number of occurrences of proteins in functions or pathway annotations stored in the Ingenuity Pathways Knowledge Base [21]. The whole Ingenuity Knowledge Base was considered as reference set.

## Results

### Two-dimensional electrophoresis reveals differential protein expression kinetics after in vivoinfection with either virulent E75 or attenuated E75CV1 ASFV-isolates

To widen and deepen our understanding of the mechanisms underlying ASFV-pathogenesis and immune response to ASFV, we characterized the variation in GLN protein profiles of pigs infected with E75 (high virulence) and E75CV (low virulence) at different times post-infection using a 2-DE proteomics approach. Our previous study [16] allowed to verify homogeneity between animals and that no outlier there was among the pigs studied. In these conditions, the technical variation can be the dominate source of variation [23]. To overcome the inherent experimental variations of this technique, we performed simultaneously four replicate (four gels) of each day and condition and more than 85% of the spots were common between gels. After image analysis and visual confirmation of gels, around 800 spots were detected. Paired analyses between infected and control groups detected 80, 94, 62 and 39 differently changed spots with E75CV1 attenuated strain at 1, 3, 7 and 31 dpi respectively, corresponding to 42, 72, 53 and 33 different proteins. With E75 virulent isolate 57, 83 and 34 spots result differentially expressed at 1, 3 years, 7 dpi, corresponding to 33, 60 and 24 different proteins. In some cases, multiple spots were unambiguously identified as the same protein, it could arrive due to post-translational modification (crucial in the control of numerous regulatory pathways, degradation of proteins, biochemical alterations or pathogenesis), different isoforms derived from different genes of a multigen family, proteolytic damage or chemical modification of protein during sample preparation.

Differences in protein profiles were not only observed between virulent and attenuated ASFV strains, but also were shown between the different times post-infection tested. The most dramatic changes found affected lymph nodes from pigs infected with E75 at day 7pi, coinciding with the late phase of the E75 lethal virus infection, probably reflects the massive tissue destruction observed at this time post-infection, with only a few intact cells being present, mostly corresponding to infiltrates of ASFV-infected macrophages [16]. GLNs from day 7 E75-infected pigs showed most proteins downregulated while infection with the attenuated E75CV1 virus upregulates a larger number of proteins than the virulent E75 strain at days 1, 3 and 7 pi, respectively (Table 1). As expected, there are a considerable number of spots in common that change after infection with the two isolates (36, 62 and 23 at 1, 3 and 7 dpi respectively), while the rest became altered only during the infection with one of the isolates (Additional files 1, 2, 3, 4 show the changes of expression of all proteins altered after infection and additional MS/MS information is available in Ref. [24]). The proteins with the largest change in fold change are shown in Tables 2, 3, 4, 5. Similarly, the expression of some proteins became apparent at specific times post-infection, while others were affected throughout the infection. As good examples, albumin and cytoskeletal proteins such as actin-like proteins were commonly affected by both ASFV strains, while many other proteins were differentially affected, such as heat shock protein B1 (HSPB1), involved in stabilizing actin filaments after stress, that results up-regulated exclusively at 1 dpi with E75CV1 [25]. Of special interest were the proteins differentially regulated after the infection with the E75CV1 attenuated strain, reflecting changes in the immune system. Interestingly, some of these proteins became upregulated as soon as day 1 pi with E75CV1 (Additional file 1), including proteasome activator complex (PSME 1 and 2). Two proteins were inversely regulated after infection with the attenuated E75CV1 strain: HSPA1B and vimentin. While HPSA1B showed a downregulation, vimentin was upregulated at day 1 pi. Other proteins specifically upregulated at day 1 pi with E75CV1 were HSPB1, enolase or lymphocyte cytosolic protein 1 (LCP-1) (Additional file 1). As expected, the early events described at day 1 pi with E75CV1, correlate with the profile of upregulated proteins specifically found by day 31 pi and related with the immune response (Additional file 4), including immunoglobulin component fractions, such as IGKC and IGHG3, or components of the proteasome such as PMSA1 and PMSA6. The up-regulation of retinol-binding protein 4 (responsible for retinol transport), galactose mutarotase or calreticulin are also of interest.

**Table 1:** **Number of regulated spots in porcine lymph node at different times post-infection**

	1 dpi		3 dpi		7 dpi		31 dpi	
E75CV1/control (attenuated)	51 ↑	29 ↓	49 ↑	45 ↓	28 ↑	34 ↓	35 ↑	4 ↓
E75/control (virulent)	32 ↑	25 ↓	30 ↑	53 ↓	1 ↑	33 ↓	–	–

**Table 2:** **The top proteins with the largest change in expression in response to ASFV infection (1 dpi)**

							Attenuated-control	
ID SSP	ID	Gen name	Protein name	p*I*	Mw (kDa)	% coverage	Fold change	*p* value
5605	F1RUN2	ALB	Serum albumin Sus scrofa	5.98	71.60	19	34.7	0.0006
605	P02543	VIM	Vimentin Sus scrofa	5.06	53.70	63	27.5	0.001
1307	Q6QAQ1	ACTB	Actin.cytoplasmic 1 Sus scrofa	5.29	42.1	19	11	0.0002
6718	P02554	TUBB	Tubulin beta chain Sus scrofa	4.78	50.30	17	10.1	0.0024
8411	I7GKE9	EEF2	Similar to human eukaryotic translation elongation factor Macaca fascicularis	5.93	36.4	21	6.7	0.002
4807	F1RMN7	HPX	Hemopexin Sus scrofa	6.59	52.10	21	4.5	0.0003
8315	F1RS36	HSPA5	78 kDaglucose-regulated protein Sus scrofa	5.21	70.3	24	4.2	0.0175
8219	I3L816	HNRNPH1	Heterogeneous nuclear ribonucleoprotein H Sus scrofa	6.44	46.6	34	3.8	0.0014
8107	K7EJP1	ATP5A1	ATP synthase subunit alpha.mitochondrial Homo sapiens	5.51	15.40	29	3.5	0.0047
9602	I3LK59	ENO	Enolase Sus scrofa	8.93	38.20	29	3.4	0.0005
4806	Q6S4N2	HSPA1B	Heat shock 70 kDa protein 1B Sus scrofa	5.6	70.30	18	− 23.1	0
9508	I3LEC2	PCBP1	Poly(rC)-binding protein 1 Sus scrofa	6.66	37.9	39	− 15	0.0001
7420	F1RKU0	IDH3A	Isocitrate dehydrogenase [NAD] subuni talpha, mitochondrial Sus scrofa	6.72	40.1	24	− 10	0.0105
407	F1M0S3	TPM2	Tropomyosin beta chain Rattus norvegicus	5.19	31.1	38	− 9.6	0.0003
502	C9J9K3	RPSA	40S ribosomal protein SA Homo sapiens	5.15	30	62	− 8.6	0.0001
302	P62258	YWHAE	14-3-3 protein epsilon Homo sapiens	4.79	29.2	44	− 7.5	0.0023
9804	P09571	TF	Serotransferrin Sus scrofa	6.93	78.90	36	− 6.9	0.0013
9401	P00355	GAPDH	Glyceraldehyde-3-phosphate dehydrogenase Sus scrofa	8.51	36.1	34	− 5.7	0.001
205	F2Z558	YWHAZ	14-3-3 protein zeta Sus scrofa	4.77	28.20	42	− 5.4	0.0032
9107	F1S3U9	PRDX1	Peroxiredoxin 1 Sus scrofa	8.67	22.1	44	− 5.1	0.0052

							Virulent-control	
ID SSP	ID	Gen name	Protein name	p*I*	Mw (kDa)	% coverage	Fold change	*p* value
4107	F1RUN2	ALB	Serum albumin Sus scrofa	5.98	71.60	5	13.4	0.0012
6718	P02554	TUBB	Tubulin beta chain Susscrofa	4.78	50.30	17	11.3	0.0003
5411	F1RPH0	PGK1	Phosphoglycerate kinase Sus scrofa	6.32	43.4	30	7.3	0.0009
8214	D0G7F6	TPI1	Triosephosphate isomerase Sus scrofa	6.54	23.90	89	6.5	0.001
8411	I7GKE9	EEF2	Similar to human eukaryotic translation elongation factor Macaca fascicularis	5.93	36.4	21	6.5	0.0023
4308	B6VNT8	ACTC1	Alpha actin 1 Sus scrofa	5.23	42.3	8	5	0.0005
8107	K7EJP1	ATP5A1	ATP synthase subunit alpha.mitochondrial Homo sapiens	5.51	15.40	29	3.5	0.0002
8219	I3L816	HNRNPH1	Heterogeneous nuclear ribonucleoprotein H Sus scrofa	6.44	46.6	34	3.4	0.0008
1410	Q9N0Y9|	TMOD3	Ubiquitous tropomodulin U-Tmod Sus scrofa	4.98	39.7	31	2.4	0.0002
7504	F1SVB0	CAPG	Macrophage-capping protein Sus scrofa	5.88	39.2	23	2.3	0.0022
9107	F1S3U9	PRDX1	Peroxiredoxin 1 Sus scrofa	8.67	22.1	44	− 6.1	0.0009
302	P62258	YWHAE	14-3-3 protein epsilon Homo sapiens	4.79	29.2	44	− 13.6	0.0027
8806	P09571	TF	Serotransferrin Sus scrofa	6.93	78.9	32	− 11.7	0.0001
301	Q5ISS9	YWHAQ	14-3-3 protein theta isoform Sus scrofa	4.7	29.20	49	− 11.4	0.0002
7715	F1RFN9	FSCN1	Fascin Sus scrofa	6.04	55.20	26	− 9.8	0.001
8404	A2A6G8	Lasp1	LIM and SH3 domain protein 1 Mus musculus	9.14	12.1	50	− 7.5	0.0004
8510	I3LDC7|	IDH1	Isocitrate dehydrogenase [NADP] (Fragment) Sus scrofa	7.64	48.7	24	− 7	0.0011
407	F1M0S3	TPM2	Tropomyosin beta chain Rattus norvegicus	5.19	31.1	38	− 5.2	0.0007
502	C9J9K3	RPSA	40S ribosomal protein SA Homo sapiens	5.15	30	62	− 4.8	0.0002
5113	Q08024	CBFB	Core-binding factor subunit beta Mus musculus	5.59	22.2	31	− 4.4	0.0008

**Table 3:** **The top proteins with the largest change in expression in response to ASFV infection (3 dpi)**

							Attenuated-control	
ID SSP	ID	Gen name	Protein name	p*I*	Mw (kDa)	% coverage	Fold change	*p* value
7226	O15144	ARPC2	Actin-related protein 2/3 complex subunit 2 Homo sapiens	6.84	34.4	45	13.1	0.0017
5435	P50395	GDI2	Rab GDP dissociation inhibitor beta Sus scrofa	6.31	50.70	49	12.4	0.0077
1427	Q0QEN7	ATP5B	ATP synthase subunit beta Sus scrofa	4.99	47	58	10.7	0.0121
1133	P20700	LMNB1	Lamin-B1 Homo sapiens	5.11	66.40	14	8.5	0.0103
3625	P11142	HSP7C	Heat shock cognate 71 kDa protein Homo sapiens	5.37	70.80	27	8.4	0.0052
8533	Q6MZU6	IGHM	IgG heavy chain Sus scrofa	6.82	52.90	25	6.1	0.0247
8321	B1ALA9	PRPS1	Phosphoribosyl pyrophosphate synthetase 1 Rattus norvegicus	7.62	24.40	37	5.6	0.0003
2322	P04899	GNA12	Guanine nucleotide-binding protein G(i) subunit alpha-2 Sus scrofa	5.35	41.00	52	5.4	0.0122
2548	P54920	NAPA	Alpha-soluble NSF attachment protein Homo sapiens	5.23	33.66	65	5.2	0.0021
6128	Q8MJ14	GPX1	Glutathione peroxidase 1 Sus scrofa	6.73	22.40	22	5.1	0.0013
2026	O89052	TUBA1B	Alpha-tubulin Mus musculus	4.85	11	60	− 34.8	0.0008
1021	P08835	ALB	Serum albumin Sus scrofa	6.08	69.70	18	− 24.3	0.0000
2131	P84856	ACTB	Actin.cytoplasmic 1 Cercopithecus pygerythrus	5.55	40.4	21	− 18.1	0.0104
4017	P61981	YWHAG	14-3-3 protein gamma Homo sapiens	4.8	28.30	47	− 17.3	0.0210
3017	P63104	YWHAZ	14-3-3 protein zeta/delta Homo sapiens	4.73	27.90	46	− 16.5	0.0001
4135	P00829	ATPB	ATP synthase subunit beta.mitochondrial Bos taurus	5.15	56.2	29	− 11.2	0.0043
4013	P31946	YWHAB	14-3-3 protein beta/alpha Homo sapiens	4.76	28.20	63	− 8.9	0.0000
4234	Q9MYP6	HSD17B14	17-beta-hydroxysteroid dehydrogenase 14 Bos taurus	6.19	28.40	7	− 5.9	0.0070
8148	P09571	TF	Serotransferrin Sus scrofa	6.93	76.90	3	− 4.9	0.0015
1018	Q2HJ57	COTL1	Coactosin-like protein Bos taurus	5.1	16.00	36	− 4.6	0.0199

							Virulent-control	
ID SSP	ID	Gen name	Protein name	p*I*	Mw (kDa)	% coverage	Fold change	*p* value
5435	P50395	GDI2	Rab GDP dissociation inhibitor beta Sus scrofa	6.31	50.70	49	16.3	0.0108
5142	P19133	FTL	Ferritin light chain Sus scrofa	5.8	28.70	32	11.6	0.0001
7226	O15144	ARPC2	Actin-related protein 2/3 complex subunit 2 Homo sapiens	6.84	34.4	45	8.7	0.0036
1427	Q0QEN7	ATP5B	ATP synthase subunit beta Sus scrofa	4.99	47	58	8.6	0.0000
1133	P20700	LMNB1	Lamin-B1 Homo sapiens	5.11	66.40	14	6.3	0.0004
7137	P60900	PSMA6	Proteasome subunit alpha type-6 Homo sapiens	6.34	27.30	26	5.1	0.0004
1640	P01009	SERPINA1	Alpha-1-antitrypsin Sus scrofa	5.54	47.4	11	4.2	0.0029
6332	P56471	IDH3A	Isocitratedehydrogenase [NAD] subunitalpha Sus scrofa	6.72	40.10	50	4.0	0.0003
1130	P52552	PRDX2	Peroxiredoxin-2 Sus scrofa	4.66	14.10	36	3.6	0.0000
1224	P08758	ANXA5	Annexin A5 Homo sapiens	4.94	35.8	59	3.2	0.0034
3123	P84856	ACTB	Actin.cytoplasmic 1 Cercopithecus pygerythrus	5.55	40.4	29	− 35.6	0.0082
4017	P61981	YWHAG	14-3-3 protein gamma Homo sapiens	4.8	28.30	47	− 19.8	0.0190
1021	P08835	ALB	Serumalbumin Sus scrofa	6.08	69.70	18	− 12.7	0.0000
2026	O89052	TUBA1B	Alpha-tubulin Mus musculus	4.85	11	60	− 11.9	0.0001
4013	P31946	YWHAB	14-3-3 protein beta/alpha Homo sapiens	4.76	28.20	63	− 11.1	0.0000
1331	Q9N0Y9	TMOD3	Tropomodulin 3 Sus scrofa	4.98	39.70	21	− 10.5	0.0051
4234	Q9MYP6	HSD17B14	17-beta-hydroxysteroid dehydrogenase 14 Bos taurus	6.19	28.40	7	− 9.8	0.0062
3017	P63104	YWHAZ	14-3-3 protein zeta/delta Homo sapiens	4.73	27.90	46	− 7.3	0.0000
515	P13489	RNH1	Ribonuclease inhibitor Sus scrofa	4.76	50.70	64	− 5.7	0.0162
1018	Q2HJ57	COTL1	Coactosin-like protein Bos taurus	5.1	16.00	36	− 4.6	0.0160

**Table 4:** **The top proteins with the largest change in expression in response to ASFV infection (7 dpi)**

							Attenuated-control	
ID SSP	ID	Gen name	Protein name	p*I*	Mw (kDa)	% coverage	Fold change	*p* value
1509	Q0QEM6	ATP5B	ATP synthase subunit beta Sus scrofa	4.99	47.10	72	26.6	0.0236
1405	F8VYX6	TUBB	Tubulin beta chain Homo sapiens	5.1	48.90	37	9.1	0.0222
8512	F1RFI1	TUFM	Elongation factor Tu Sus scrofa	6.72	49.70	46	6	0.0004
8606	P01790	IGH	Ig heavy chain V region Mus musculus	8.01	13.70	24	5.7	0.0232
1612	P02543	VIM	Vimentin Sus scrofa	5.06	53.70	57	4.5	0.0097
3319	B4DWA6	CAPZB	Highly similar to F-actin capping protein subunit beta Homo sapiens	5.77	37.8	31	4.1	0
4005	Q2TBX5	SSR4	Translocon-associated protein subunit delta Bos taurus	5.49	19.00	37	3.9	0.095
7412	Q9GKX6	GALM	Aldose 1-epimerase Sus scrofa	6.31	38.00	30	3.7	0.0044
8209	B5APU7	ARPC2	Actin-related protein 2/3 complex subunit 2 Sus scrofa	6.84	34.3	63	3.7	0.025
7108	D6RBM0	HNRNPH1	Heterogeneous nuclear ribonucleoprotein H Homo sapiens	6.97	24.2	62	3.7	0.0033
3005	B3KWQ3	ACTG	Highly similar to Actin. cytoplasmic 2 Homo sapiens	5.2	28.50	29	− 12	0.0055
7309	F1RUN2	ALB	Serum albumin Sus scrofa	5.98	71.60	30	− 5	0.0004
3720	Q5T6W5	HNRNPK	Heterogeneous nuclear ribonucleoprotein K Homo sapiens	5.46	47.70	41	− 4.9	0.018
1308	A5D989	EEF1D	Elongation factor 1-delta Bos taurus	4.94	31.2	27	− 4.8	0
3723	F1MUZ9	HSPD1	60 kDa chaperonin Bos taurus	5.71	61.10	42	− 4.3	0.0017
3318	P11493|	PPP2CB	Serine/threonine-protein phosphatase 2A catalytic subunit beta isoform Homo sapiens	5.46	34.1	35	− 3.2	0.023
4112	Q1W2K3	PSMB10	Proteasome subunit beta Sus scrofa	6.09	29.20	23	− 3.2	0.0004
2613	F1RG16	HNRNPF	Heterogeneous nuclear ribonucleoprotein F Sus scrofa	5.32	45.90	42	− 3.1	0.0452
305	I3L813	PCNA	Proliferating cell nuclear antigen Sus scrofa	4.57	29.1	63	− 2.9	0.005
2309	G7P8A3	EGM_15385	Serine/threonine-protein phosphatase Macaca fascicularis	5.55	33.9	46	− 2.4	0.0003

							Virulent-control	
ID SSP	ID	Gen name	Protein name	p*I*	Mw (Kda)	% coverage	Fold change	*p* value
3516	F1MRD0	ACTB	Actin.cytoplasmic 1.Bos taurus	5.16	42.20	38	6.1	0.0255
7309	F1RUN2	ALB	Serumalbumin Sus scrofa	5.98	71.60	30	− 26	0.011
3005	B3KWQ3	ACTG	Highly similar to Actin. cytoplasmic 2 Homo sapiens	5.2	28.50	29	− 24	0.0214
6417	Q8IY98	ACTR2	Actin-related protein 2 Homo sapiens	5.77	39.90	43	− 6.4	0.0004
6313	O88550	CASP7	Caspase 7 Rattus norvegicus	5.53	34.9	17	− 5.5	0.0125
2012	Q0IIA3	SRI	Sorcin Bos taurus	5.11	20.60	20	− 4.7	0.014
9012	Q9BGI4	PRDX1	Peroxiredoxin 1 Bos taurus	8.81	22.4	40	− 4.6	0.0013
3314	Q15181	PPA1	Inorganic pyrophosphatase Homo sapiens	5.54	33.1	41	− 4.4	0.0374
2613	F1RG16	HNRNPF	Heterogeneous nuclear ribonucleoprotein F Sus scrofa	5.32	45.90	42	− 4.3	0.0357
207	P61981	YWHAG	14-3-3 protein gamma Homo sapiens	4.8	28.4	59	− 3.4	0.017
2309	G7P8A3	EGM_15385	Serine/threonine-protein phosphatase Macaca fascicularis	5.55	33.9	46	− 2.8	0.0223
6416	Q99LC3	NDUFA10	NADH dehydrogenase [ubiquinone] 1 alpha subcomplex subunit 10. Mus musculus	7.63	40.80	22	− 2.3	0.027
2208	F2Z5C1	ANXA5	Annexin Sus scrofa	4.95	33.2	59	− 2.1	0.0313
305	I3L813	PCNA	Proliferating cell nuclear antigen Sus scrofa	4.57	29.1	63	− 2	0.0401
2108	F1SQW8	ARHGDIB	Rho protein dissociation inhibitor homolog Sus scrofa	5.08	22.90	55	− 1.8	0.047
306	F1M0S3	TPM2	Tropomyosin beta chain Rattus norvegicus	5.19	31.1	40	− 1.8	0.0155
7213	K7D9V9	HNRNPH3	Heteroproteinous nuclear ribonucleoprotein H3 Pan troglodytes	6.36	35.2	68	− 1.7	0.0419

**Table 5:** **The top proteins with the largest change in expression in response to E75CV1 infection (31 dpi)**

							Attenuated-control	
ID SSP	ID	Gen name	Protein name	p*I*	Mw (Kda)	% coverage	Fold change	*p* value
4611	Q4V7C7	ACTR3	Actin-related protein 3 homolog Rattus norvegicus	5.61	47.6	32	10.1	0.0001
7219	Q45FY6	HPRT1	Hypoxanthine–guanine phosphoribosyl transferase Sus scrofa	6.3	24.80	61	6	0.0383
1903	A0A024R972	LAMC1	Laminin. gamma 1.isoform CRA_a Homo sapiens	4.94	17.90	11	5.8	0.0189
7518	P08835	ALB	Albumin Sus scrofa	5.92	71.40	37	4.8	0.024
5513	P00348	HADH	Hydroxyacyl-coenzyme A dehydrogenase Sus scrofa	9.02	34.20	32	3.8	0.0255
3211	Q06AS6	GNAI2	Guanine nucleotide binding protein. alpha inhibiting activity polypeptide 2 Sus scrofa	5.35	41	64	3.2	0.0035
8412	P60901	PSMA6	Proteasome subunit alpha type-6 Rattus norvegicus	6.34	27.80	51	2.5	0.0193
3410	A0PA01	SERPINB9	Serpin peptidase inhibitor.clade B (ovalbumin).member 9 Sus scrofa	5.37	42.8	61	2.4	0.0137
7212	P04431	IGKC	Immunoglobulin kappa light chain Sus scrofa	8.65	12.20	39	2.4	0.0081
2514	P60953	CDC42	Chain B. Structure Of The Complex Between Dock9 and Cdc42. Homo sapiens	6.3	21.50	45	2.3	0.0019
7220	P31943	HNRNPH	Heterogeneous nuclear ribonucleoprotein H Homo sapiens	5.89	49.50	36	2.2	0.027
6112	P01860	IGHG3	Ig gamma 3 chain constant region. Sus scrofa	7.25	36.50	28	2.2	0.0055
5119	P80031	GSTP1	Glutathione S-transferase P1 Sus scrofa	8.07	23.7	46	2.1	0.0089
7214	Q9GKX6	GALM	Galactose mutarotase Sus scrofa	6.31	38.00	44	1.9	0.019
7614	P52193	CALR	Calreticulin Bos taurus	4.31	46.50	46	1.8	0.0334
7514	P01834	IGKC	Ig kappa light chain Sus scrofa	8.65	12.10	41	1.8	0.0061
5118	P27485	RBP4	Retinol-binding protein 4 Sus scrofa	5.41	23.4	19	1.8	0.0205
1711	P08670	VIM	Vimentin Homo sapiens	5.03	53.7	58	− 3.3	0.0109
8213	B8XSK0	CPNE1	Copine 1 Sus scrofa	5.43	59.6	26	− 2.6	0.0094
1611	Q5S1U1	HSPB1	Heat shock protein beta-1 Sus scrofa	6.23	22.90	16	− 2.3	0.0064

### Functional analysis of differentially expressed proteins after in vivo infection with either virulent E75 or attenuated E75CV1 ASFV-isolates

Bioinformatic tools were employed to biologically interpret the data set of protein obtained from 2-DE analysis, with the aim of gaining an insight into biological functions and pathways associated with the proteome response of porcine GLN to ASFV, as well as to discover differences in processes that occur after infection with each one of the ASFV-strains. Therefore, we have analyzed our data set using IPA, focused on cell functions and on canonical pathways, so-called because they contain well-established knowledge about specific relationships between groups of proteins. Differentially expressed proteins were involved in different aspects of the host–pathogen interaction (Additional files 5, 6, 7, 8, 9, 10, 11). With respect to functions, inflammatory and immunological disease were functions altered by both virus, but a greater number of proteins involved in these functions were differentially expressed after E75CV1 than E75 infection, at 1 dpi. Thus, we have found the up-regulation of proteins such as: HSPA5 and HSPB1, LCP1, vimentin, C reactive protein or hemopexin, a protein that positively regulates the interferon-gamma-mediated signaling pathway [26]. Accordingly, our previous qPCR results [16] showed an over-expression of interferon-gamma after infection with E75CV1 but not due to E75 infection, at 1 dpi. Conversely, GLNs from E75-infected pigs showed, at day 1 pi, the specific downregulation of SERPINA3, an acute phase protein that is induced during inflammation [27] or vitamin D-binding protein (implicated in macrophage activation and inflammation) [28], most probably contributing to a delayed inflammatory response to the virulent isolate.

**Figure 1:**
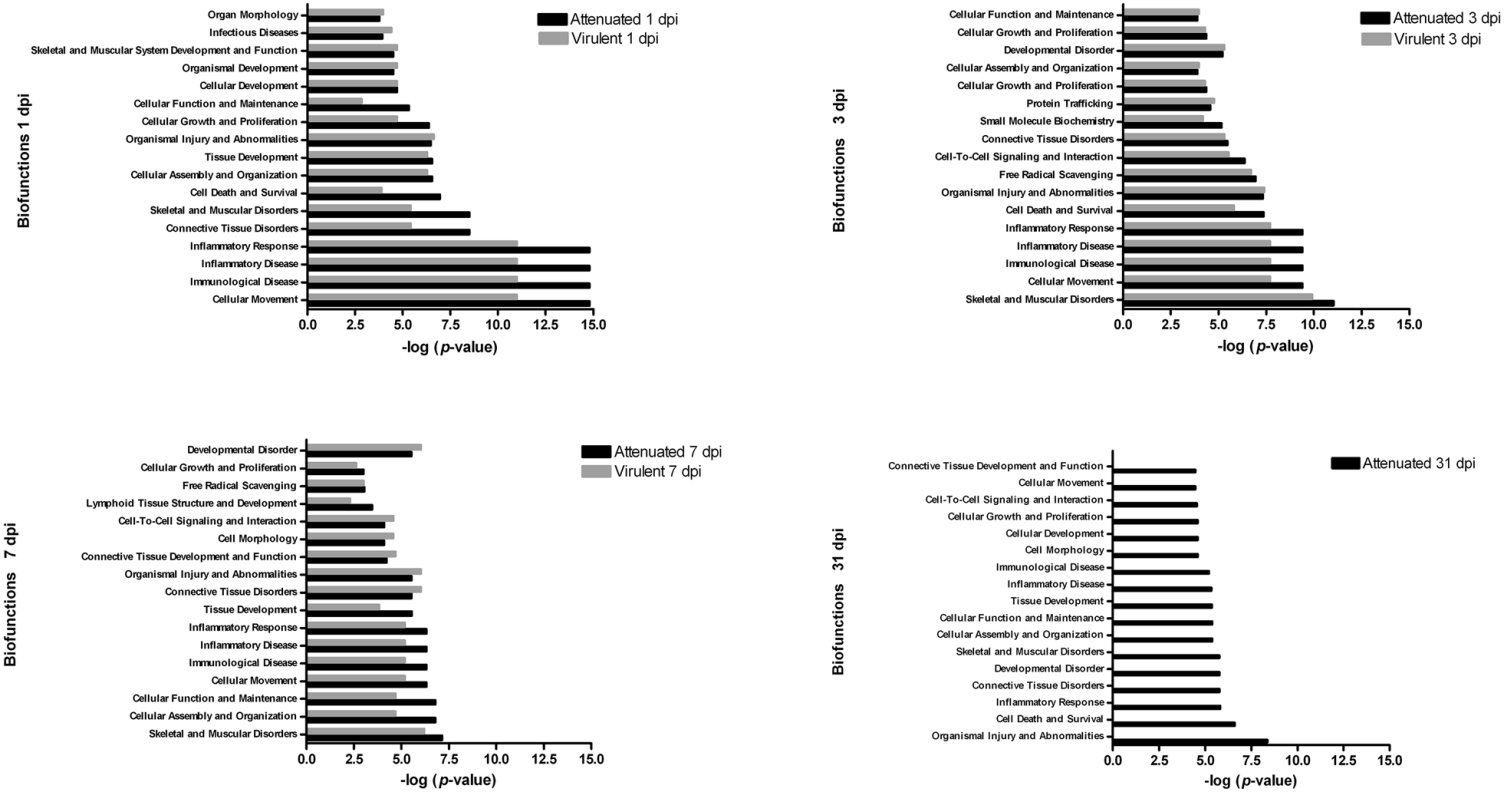
**Enriched functions associated with the response of porcine lymph node to ASFV during infection.** The analysis is derived from the Ingenuity Pathways Analysis.

Figure 1 also included other functions (derived from the Ingenuity Pathways Analysis) significantly altered at early times after infection with both virus strains, e.g. those associated to free radical scavenging, cell death and cell-to-cell signaling and interaction, while cellular assembly and organization or cellular functions showed fewer modifications. Nevertheless, by day 7 pi with E75CV1, coinciding with the recovery of the infected pigs [16], these latter functions became more relevant. Conversely, GLN from E75-infected pigs by day 7 pi, showed protein profiles enriched in functions related to tissue destruction when compared to E75CV1; these were mainly associated with connective tissue disorders, skeletal and muscular disorders or with organismal injury and abnormalities. Some of these abnormalities became evident as early as at day 3 pi, coinciding with the ASFV replication in the GLN [16].

The association of the proteins significantly affected by ASFV infection with canonical pathways is shown in Table 6. As expected, the remodelling of epithelial adherent junction pathways was enriched for both viruses. In this canonical pathway, several members of the actin family participate, shown in here as differentially regulated. Also, clathrin-mediated endocytosis signaling pathways, involving molecules such as actin, heat shock proteins, serpin or transferrin, were regulated at all times post-infection with the exception of day 1 pi with E75CV1. One of the most remarkable and novel observations of our work affects the 14-3-3 mediated signaling, inhibited by both isolates at 1 and 3 dpi. It is also worth highlighting the differential regulation of Rho GTPases in GLNs from E75CV1 infected pigs between days 1 and 7, coinciding with the induction of innate immunity and the resolution of the subclinical ASFV infection [29]. Regarding pathways regulated by only one virus, or in some cases at only times post-infection, is worthy to mention the G2/M DNA damage checkpoint regulation, involving the different 14.3.3 isoforms which we have been found downregulated after infection.

**Table 6:** **Analysis of the canonical pathways corresponding to the obtained data set**

	14-3-3-mediated signaling	Remodeling of epithelial adherens junctions	Clathrin-mediated endocytosis signaling	Signaling by rho family GTPases	RhoGDI signaling	Epithelial adherens junction signaling	Regulation of actin-based motility by rho	Cell Cycle: G2/M DNA damage checkpoint regulation	Mechanisms of viral exit from host cells	Death receptor signaling	Virus entry via endocytic pathways	Myc mediated apoptosis signaling
E75CV1 (dpi)												
1	X	X	–	–	X	–	X	X	–	–	–	–
3	X	X	X	–	X	–	–	X	–	–	–	–
7	–	X	X	X	X	X	–	–	–	–	–	–
31	–	X	X	X	X	–	X	–	–	–	–	–
E75 (dpi)												
1	X	X	X	–	–	X	–	–	X	–	X	–
3	X	X	X	–	–	X	–	X	–	–	–	X
7	–	X	X	–	X	X	–	–	–	X	–	–

The analysis is derived from the Ingenuity Pathways Analysis (*p* < 0.05).

X: affected by ASFV infection; –: no affected by ASFV infection.

## Discussion

Proteome approaches are being increasingly used in many different systems to investigate host–microbe interactions and it has provided important information about the protein profile of cells infected with ASFV [10, 17]. The results presented here could complement the aforementioned ones since they have been performed with infected pig tissue. In this context, in vivo models could help to reflect the multiple events undergone by the host upon pathogen infection. Here, we applied a proteomics approach, based on 2-DE coupled to an in vivo experiment, to obtain new insights about the differential responses triggered by attenuated and virulent homologous ASFV strains. Lymphnodes are complex tissues composed by different cell types and, despite ASFV main targets are the macrophages, it is known that the effect of virus infection is not only restricted to the infected cell but also to the cascade of events it provokes the massive apoptosis induced in surrounding non-infected lymphocytes, this being a key event for ASF pathogenesis, responsible for lymphoid organ impairment in acute ASFV infection [30]. Besides, the lesions found in ours previous manuscript [16] after necropsy together with a comparative transcription profile of key immune mediators that were differentially modulated throughout the infections point gastrohepatic lymph node as the organ of choice to improve our knowledge about of the differential porcine response against virulent or attenuated ASFV isolated, to further compare globally the immunopathogenesis of both virus strains.

Also, we used IPA in a hypothesis generative manner aiming to unmask the most relevant functions and pathways altered throughout the infection in GLN, one of its main target organs. Overall, the analysis shows that, together with pathways involved in host-immune responses, a significant number of other host functions are modified, demonstrating the complex host-virus interactions that occur in vivo. Despite the much lower ASFV load observed after infection with the attenuated ASFV strain [16], major protein changes were detected early after infection with E75CV1, affecting not only the infected macrophages, but also surrounding cell-types in an indirect manner. Therefore, E75 and E75CV1 not only differed in the kinetics and in the clinical symptoms they provoke, but also in the pathways activated throughout the infections. The fewer number of proteins with virulent isolated at 1 dpi might help explain the failure of the innate immune system to detect and control the first rounds of E75-replication, thus allowing its rapid spread. Conversely, efficient regulation of the innate immune system became evident for E75CV1 as early as at 1 day pi. As example, the implication of the Rho GTPases signalling pathway by E75CV1 throughout infection, (Table 6), perhaps confirming the relevance of this pathway in the innate immune system. Although the best-known function of this protein family is regulate and coordinates of actin and microtubule cytoskeletal dynamics and adhesion [31], Rho GTPases regulate numerous basic cell functions including regulation of the signaling pathways and cellular responses that enable to phagocytes perform their innate immune functions to respond invading pathogens. Thus, they are key regulators of cell migration (through various cell-surface receptors as TLRs), reactive oxygen species (ROS) production by NADPH oxidase, phagocytosis and degranulations well as a essential, and perhaps unique, roles in the motile responses of leukocytes [32]. Another studies also indicate that Rho GTPases provide alternative pathways to regulate NF-kB transcriptional activity in cells of the innate immune system [32].

Several other proteins became differentially regulated by day 1 pi, as vimentin, enolase, HSPB1 or lymphocyte cytosolic protein 1 (LCP-1) (Additional file 1) from E75CV1-pigs. Vimentin has been described as an important molecule during ASFV morphogenesis by changing its localization to viral factories after in vitro infection [33]. Thus, the upregulation of vimentin, at day 1 pi, might be an indirect effect provoked by E75CV1 infection on surrounding non-infected cells. Coincidently, vimentin and other proteins specifically upregulated at day 1 pi with E75CV1, such as HSPB1, enolase or LCP-1 (Additional file 1), share both their potential role in immune defense and their description as autoantigens in autoimmune disorders [34]. In particular, the role of HSPs as immunogenic molecules able to activate T cells has been long known [35] and several reports have shown that pretreatment with HSPs protects from autoimmune disease [36]. Also, T-cell response to α-enolase could be involved in the pathogenesis of autoimmune diseases [37]. We are currently trying to unmask the potential presence of auto-antibodies in pigs infected with E75CV1 by using sera from day 31 pi and the upregulated autoantigens found at day 1 pi. These markers might be of utility to diagnose chronic infections and/or to better understand ASFV pathogenesis during chronic infections.

Of particular interest could be the complex regulation found for several members of the heterogeneous nuclear ribonucleoprotein (hnRNP) complex, RNA-binding proteins involved in downstream gene regulation and G2/M DNA damage checkpoint regulation, apoptosis and immune regulation [38]. hnRNPH1, a protein previously shown as being involved in virus replication [39], was upregulated at day 1 pi with both viruses, overexpression that was maintained for the attenuated E75CV1 virus at day 3 and 7 pi. It has been reported that upregulation of hnRNPH causes a decrease in HIV virion production [40]. Interestingly, it is worth noting the involvement of hnRNPH expression with TNF-α production [41] and NF-κB activation, both proteins being involved in inflammation and immune response. In Hepatitis C virus infection, it has been proposed that hnRNP might limit the amount of viral RNA genomes available for incorporation into virus [42]. These changes in expression could be significant and add information to the only described interaction between the ASFV p30 antigen and hnRNPK in Vero infected cells [43]. The authors suggest that the interaction hnRNPK-p30 could contribute to the host cell shut-off and represent a possible additional mechanism by which ASFV down-regulates host cell mRNA translation.

Together with pathways involved in immune responses, it is worth highlighting, the pathways involved in cytoskeletal and epithelial adherent junction remodeling (Table 6), reflecting the use that ASFV makes of cytoskeleton from virus entry to virus morphogenesis and cell egress [9, 44]. This result perfectly correlates with the function that this pathway plays during ASFV entry in pig cells [45], involving molecules such as actin, heat shock proteins, serpin or transferrin, which have been found altered in our study. Similarly, the clathrin-mediated endocytosis pathways here highlighted, have already been described as essential for ASFV entry in susceptible cells [45], giving consistency and validity to our results. Clathrin-mediated endocytosis, a strategy used by many viruses for cell entry [46], is commonly activated during both attenuated and virulent ASFV infection (Table 6) from day 1 pi with E75 and from day 3 pi with the E75CV1 strain, coinciding with the differential kinetics of ASFV in vivo replication observed for both strains [16]. The differential expression of several other proteins also involved in cytoskeleton formation it is worthy to be discussed. Thereby, the Rho GTPase family is also involved in the regulation of microtubules during dynein-mediated capsid transport of herpes virus associated Kaposi’s sarcoma [47], a pathway also required for ASFV entry, morphogenesis and exit from the infected cells [31, 44]. The inhibition of RhoGTPases by RhoGDI by attenuated isolate might have a negative effect in all these processes thus impairing the in vivo transmission of E75CV1. Conversely, the down-expression of RhoGDI at 7 dpi, might contribute to the successful systemic dissemination of E75. Also, the results observed with RP/EB microtubule-associated protein (which negatively regulates microtubule formation) point in this direction. Thus, interestingly, in our study this protein is down-regulated at 1 dpi with both virus (which would facilitate the formation of the microtubules and therefore traffic virus at the onset of infection) but over-expressed to 3 dpi only with attenuated isolate (inhibiting the formation of the microtubules and thus the transport of the attenuated isolate).

A chapter apart deserves discussing the sub-expression found at early times post-infection (day 1 and 3 pi) with both viruses, of several of the 7 isoforms of 14-3-3 protein, a novel finding for ASFV. 14-3-3 interactome studies have demonstrated that 14-3-3 proteins participate in many events associated with infection in other viruses [48], mainly activated by dsRNA. The downregulation observed for both E75CV1 and E75 in vivo might reflect the need of ASFV to evade the innate immune responses triggered by 14-3-3, including the activation of TNF and NFκB signalling or any other antimicrobial responses triggered by activating the TLR-14-3-3 pathways [49]. While 14-3-3 has been strongly associated with the innate immunity activated in response to dsRNA [50], its negative effect on virus morphogenesis [51], and ASFV-exit from the cell, [52], have been also described. On the other hand, an important function of 14-3-3 proteins is to inhibit apoptosis [53] and downregulation of 14-3-3 might also have a direct effect on the G2/M DNA damage checkpoint regulation [54], a pathway that prevents cells with damaged DNA entering the M phase of cell division before repairing. So, a defective G2/M checkpoint leads DNA damaged cells to apoptosis [55]. Curiously enough, E75CV1 downregulates the G2/M cell cycle control checkpoint both at day 1 and 3 pi, while E75 does it at day 3 pi (Table 6) as described for other viruses [56]. The concomitant downregulation of the G2/M DNA damage checkpoint and 14-3-3 observed might contribute to differentially activate the apoptosis of the infected cells, perhaps contributing to the deficient in vivo dissemination of E75CV1 [7]. All together, these results seem to reflect a very complex regulation of apoptosis during ASFV infection, as has been previously postulated [57]. These results complement previous work demonstrating the effect of DNA damage and apoptosis in ASFV-in vitro replication [58], confirming the role that 14-3-3 play during apoptosis inhibition [53], as has been demonstrated for other viruses [59]. Studies to confirm the relevance of 14-3-3 in this inhibition might help to design novel antiviral strategies.

The last part of the discussion will be dedicated to the results obtained with samples harvested at day 31 after the infection with E75CV1, that might be very useful to understand the intrinsic mechanisms involved in protection against E75-virulent challenge. As expected, the proteomic analysis performed with these samples seem to confirm the key relevance that both antibodies [4] and CD8 T-cells [15, 60], play in protection against ASFV. The immunoglobulin (Ig) isoforms detected could play a dual role participating either in ASFV-antibody mediated protection [4], or also in the formation of immune complexes found in chronically ASFV infected pigs [15]. PMSA1 and PMS6 are involved in swine leukocyte antigen class I (SLAI) presentation and CD8-T cell induction but also have endoribonuclease activity, playing important defensive roles in response to external stimuli [61]. The up-regulation of other proteins with implication in the antigenic presentation, such as retinol-binding protein 4 (responsible for retinol transport) and galactose mutarotase (GALM) are also of interest. Retinol is a potent regulator of B-cell receptor function and B-cell activation [62] and regulates GALM gene expression, which could have an important role in cell adhesion and antigen presentation [63]. Other components of the B cell response were upregulated, including members of Ig family, proteins implicated in antigenic processing and SLAI antigen presentation (as proteasome subunits), calreticulin or serpin. Calreticulin (CRT) is a Ca2^+^-binding protein involved in more than 40 functions, including the unfolded protein response (UPR) and antigen presentation. Interestingly, CRT has been found upregulated in vitro as part of endoplasmic reticulum (ER) stress and the UPR provoked by ASFV in infected cell [64]. UPR is a antiviral mechanism, against which many viruses develop multiple strategies [65] and on the other hand, CRT transport antigens to the ER, facilitating antigen presentation in association with the major histocompatibility complex class I (MHC-I) molecules to elicit peptide-specific CD8^+^ T cell responses [66]. In accordance with the latter, several other proteins involved in antigen presentation and T-cell activation have been found up-regulated at day 31 pi with E75CV1, including SERPINB9 and proteasomal subunits. Serpin B anti-proteases have been defined as regulators of the immune response up-regulated during several virus infections, including HIV [67] and Epstein–Barr virus infection [68]. SERPINB9 has been shown to be involved in protection of antigen presenting cells, enhancing T cell activation and immune response [68], including in IFN-γ production and antiviral cytopathic responses [69] and survival of CD8^+^ memory T cells [70]. The proteomic data obtained with samples from E75CV1-recoverd pigs (31 dpi) perfectly fits with the increasing evidence that Th1 and specific CD8 T-cells play in protection [3], including our previous data using this same ASFV-infection model [16]. Altogether, our results could indicate that the over-expression of CRT, SERPINB9 and subunits from the proteasome reflect the relevance that SLAI presentation and CD8 T-cell activation play during ASFV infection, opening new avenues to fight the disease. Interestingly, these three components have already been used as genetic adjuvants to improve the specific immunity against several pathogens [68]. We are currently extending our studies to the field of ASFV vaccinology. Thus, today we know that targeting antigens to the proteasome improves the protection against ASFV even in the absence of antibodies [14, 60]. The knowledge gained here opens new avenues to improve these strategies in the near future.

In conclusion, the data presented here are the first to compare kinetics of protein expression profiles from pigs infected by homologous virulent or attenuated strains of ASFV, through a proteomics approach coupled with a large-scale in vivo infection, in order to allow the generation of advances in our understanding about the pig immune response to virus and pathogenesis of ASF over time. Our results confirm a differential interaction with the immune system for both viruses. Thus, GLN from E75CV1 infected pigs showed the largest number of differentially upregulated proteins as early as 1 dpi, many of them involved in the activation of different innate immune pathways, including autoantigens. In addition to a lower replication efficiency at early time post-infection by attenuated isolated, the induction of specific antibody and T-cell responses at 31 dpi, were observed, once E75CV1 has been cleared. We believe that the increased information yielded by this global approach could improve our knowledge about the major point underlying host–pathogen interactions and might be the important for the development of an efficacious ASF vaccine.

## Additional files

**Additional file 1.** **Differentially regulated proteins in response to ASFV infection (1 dpi).** Changes of expression of all proteins altered after this time after infection.

**Additional file 2.** **Differentially regulated proteins in response to ASFV infection (3 dpi).** Changes of expression of all proteins altered after this time after infection.

**Additional file 3.** **Differentially regulated proteins in response to ASFV infection (7 dpi).** Changes of expression of all proteins altered after this time after infection.

**Additional file 4.** **Differentially regulated proteins in response to ASFV infection (31 dpi).** Changes of expression of all proteins altered after this time after infection.

**Additional file 5.** **Canonical pathways and functions significantly regulated by attenuated ASFV in porcine lymph node at 1 dpi**. The analysis is derived from the Ingenuity Pathways Analysis.

**Additional file 6.** **Canonical pathways and functions significantly regulated by virulent ASFV in porcine lymph node at 1 dpi**. The analysis is derived from the Ingenuity Pathways Analysis.

**Additional file 7.** **Canonical pathways and functions significantly regulated by attenuated ASFV in porcine lymph node at 3 dpi**. The analysis is derived from the Ingenuity Pathways Analysis.

**Additional file 8.** **Canonical pathways and functions significantly regulated by virulent ASFV in porcine lymph node at 3 dpi.** The analysis is derived from the Ingenuity Pathways Analysis.

**Additional file 9.** **Canonical pathways and functions significantly regulated by attenuates ASFV in porcine lymph node at 7 dpi.** The analysis is derived from the Ingenuity Pathways Analysis.

**Additional file 10.** **Canonical pathways and functions significantly regulated by virulent ASFV in porcine lymph node at 7 dpi**. The analysis is derived from the Ingenuity Pathways Analysis.

**Additional file 11.** **Canonical pathways and functions significantly regulated by attenuated ASFV in porcine lymph node at 31 dpi**. The analysis is derived from the Ingenuity Pathways Analysis.

## References

[1] Rock DL (2017). Challenges for African swine fever vaccine development-”… perhaps the end of the beginning.”. Vet Microbiol.

[2] Portugal R, Coelho J, Höper D, Little NS, Smithson C, Upton C, Martins C, Leitão A, Keil GM (2015). Related strains of African swine fever virus with different virulence: genome comparison and analysis. J Gen Virol.

[3] Takamatsu HH, Denyer MS, Lacasta A, Stirling C, Argilaguet J, Netherton CL, Oura C, Martins C, Rodriguez F (2013). Cellular immunity in ASFV responses. Virus Res.

[4] Escribano JM, Galindo I, Alonso C (2013). Antibody-mediated neutralization of African swine fever virus: myths and facts. Virus Res.

[5] Reis AL, Netherton C, Dixon LK (2017). Unraveling the armor of a killer: evasion of host defenses by African swine fever virus. J Virol.

[6] Zhang F, Hopwood P, Abrams CC, Downing A, Murray F, Talbot R, Archibald A, Lowden S, Dixon LK (2006). Macrophage transcriptional responses following in vitro infection with a highly virulent African swine fever virus isolate. J Virol.

[7] Ramiro-Ibañez F, Ortega A, Ruiz-Gonzalvo F, Escribano JM, Alonso C (1997). Modulation of immune cell population and activation markers in the pathogenesis of African swine fever virus infection. Virus Res.

[8] Gil S, Sepúlveda N, Albina E, Leitão A, Martins C (2008). The low-virulent African swine fever virus (ASFV/NH/P68) induces enhanced expression and production of relevant regulatory cytokines (IFNalpha, TNFalpha and IL12p40) on porcine macrophages in comparison to the highly virulent ASFV/L60. Arch Virol.

[9] Alonso C, Galindo I, Cuesta-Geijo MA, Cabezas M, Hernaez B, Muñoz-Moreno R (2013). African swine fever virus-cell interactions: from virus entry to cell survival. Virus Res.

[10] Alfonso P, Rivera J, Hernaez B, Alonso C, Escribano J (2004). Identification of cellular proteins modified in response to African swine fever virus infection by proteomics. J Proteomics.

[11] Golding JP, Goatley L, Goodbourn S, Dixon LK, Taylor G, Netherton CL (2016). Sensitivity of African swine fever virus to type I interferon is linked to genes within multigene families 360 and 505. Virology.

[12] Galindo I, Alonso C (2017). African swine fever virus: a review. Viruses.

[13] Sánchez EG, Quintas A, Nogal M, Castelló A, Revilla Y (2013). African swine fever virus controls the host transcription and cellular machinery of protein synthesis. Virus Res.

[14] Lacasta A, Ballester M, Monteagudo PL, Rodriguez JM, Salas ML, Accensi F, Pina-Pedrero S, Bensaid A, Argilaguet J, Lopez-Soria S, Hutet E, Le Potier MF, Rodriguez F (2014). Expression library immunization can confer protection against lethal challenge with African swine fever virus. J Virol.

[15] Fernandez A, Perez J, de Martin J, Carrasco L, Dominguez J, Sierra MA (1992). Localization of African swine fever viral antigen, swine IgM, IgG and C1q in lung and liver tissues of experimentally infected pigs. J Comp Pathol.

[16] Lacasta A, Monteagudo P, Jiménez-Marin A, Accensi F, Ballester M, Argilaguet J, Galindo-Cardiel I, Segalés J, Salas ML, Dominguez J, Moreno A, Garrido JJ, Rodríguez F (2015). Live attenuated African swine fever viruses as ideal tools to dissect the mechanisms involved in viral pathogenesis and immune protection. Vet Res.

[17] Rodriguez J, Salas M, Santaren J (2001). African swine fever virus induced polypeptides in porcine alveolar macrophages and in Vero cells: two-dimensional gel analysis. Proteomics.

[18] Rodrigo G, Daros JA, Elena SF (2017). Virus-host interactome: putting the accent on how it changes. J Proteomics.

[19] Guo X, Hu H, Chen F, Li Z, Ye S, Cheng S, Zhang M, He Q (2016). iTRAQ-based comparative proteomic analysis of Vero cells infected with virulent and CV777 vaccine strain-like strains of porcine epidemic diarrhea virus. J Proteomics.

[20] Sun J, Jiang Y, Shi Z, Yan Y, Guo H, He F, Tu C (2008). Proteomic alteration of PK-15 cells after infection by classical swine fever virus. J Proteome Res.

[21] Ramírez-Boo M, Nuñez E, Jorge I, Navarro P, Fernandes L, Segales J, Garrido JJ, Vázquez J, Moreno A (2011). Quantitative proteomics by 2-DE, 16O/18O labelling and linear ion trap mass spectrometry analysis of lymph nodes from piglets inoculated by porcine circovirus type 2. Proteomics.

[22] Collado-Romero M, Prado-Martins R, Arce C, Moreno A, Lucena C, Carvajal A, Garrido JJ (2012). An in vivo proteomic study of the interaction between Salmonella Typhimurium and porcine ileum mucosa. J Proteomics.

[23] Pallen C, Friry-Santini C, Herouet-Guicheney C, Capt A (2014). Technical variability of 2D gel electrophoresis—application to soybean allergens. Toxic Rep.

[24] Central Service for Research Support. http://www.uco.es/scai/geles_2017.html

[25] Wettstein G, Bellaye PS, Micheau O, Bonniaud P (2012). Small heat shock proteins and the cytoskeleton: an essential interplay for cell integrity?. Int J Biochem Cell Biol.

[26] Xu M, Tan C, Hu J, Alwahsh SM, Yan J, Hu J, Dai Z, Wang Z, Zhou J, Fan J, Huang X (2014). Expression of hemopexin in acute rejection of rat liver allograft identified by serum proteomic analysis. Shock.

[27] Janciauskiene S, Wright HT (1998). Inflammation, antichymotrypsin, and lipid metabolism: autogenic etiology of Alzheimer’s disease. BioEssays.

[28] Pacini S, Punzi T, Morucci G, Gulisano M, Ruggiero M (2012). Effects of vitamin D-binding protein-derived macrophage-activating factor on human breast cancer cells. Anticancer Res.

[29] Bidaud-Meynard A, Binamé F, Lagrée V, Moreau V (2017). Regulation of Rho GTPase activity at the leading edge of migrating cells by p190RhoGAP. Small GTPases.

[30] Ramiro-Ibañez F, Ortega A, Brun A, Escribano JM, Alonso C (1996). Apoptosis: a mechanism of cell killing and lymphoid organ impairment during acute African swine fever virus infection. J Gen Virol.

[31] Quetglas J, Hernaez B, Galindo L, Muñoz R, Cuesta M, Alonso C (2012). Small Rho GTPases and cholesterol biosynthetic pathway intermediates in African swine fever virus infection. J Virol.

[32] Bokoch GM (2005). Regulation of innate immunity by Rho GTPases. Trends Cell Biol.

[33] Stefanovic S, Windsor M, Nagata KI, Inagaki M, Wileman T (2005). Vimentin rearrangement during African swine fever virus infection involves retrograde transport along microtubules and phosphorylation of vimentin by calcium calmodulin kinase II. J Virol.

[34] Moy L, Levine J (2014). Autoimmune hepatitis: a classic autoimmune liver disease. Curr Probl Pediatr Adolesc Health Care.

[35] Zugel U, Kaufmann SH (1999). Role of heat shock proteins in protection from and pathogenesis of infectious diseases. Clin Microbiol.

[36] Fijak M, Iosub R, Schneider E, Linder M, Respondek K, Klug J, Meinhardt A (2005). Identification of immunodominant autoantigens in rat autoimmune orchitis. J Pathol.

[37] Nahm DH, Lee KL, Shin JY, Ye JM, Kang Y, Park HS (2006). Identification of alpha-enolase as an autoantigen associated with severe asthma. J Allergy Clin Immunol.

[38] Yabas M, Elliott H, Hoyne GF (2015). The role of alternative splicing in the control of immune homeostasis and cellular differentiation. Int J Mol Sci.

[39] Lee JW, Liao PC, Young KC, Chang CL, Chen SS, Chang TT, Lai MD, Wang SW (2011). Identification of hnRNPH1, NF45, and C14orf166 as novel host interacting partners of the mature hepatitis C virus core protein. J Proteome Res.

[40] Jablonski JA, Caputi M (2009). Role of cellular RNA processing factors in human immunodeficiency virus type 1 mRNA metabolism, replication, and infectivity. J Virol.

[41] Mishra KP, Shweta Diwaker S, Ganju L (2012). Dengue virus infection induces up-regulation of hn RNP-H and PDIA3 for its multiplication in the host cell. Virus Res.

[42] Poenisch M, Metz P, Blankenburg H, Ruggieri A, Lee JY, Rupp D, Rebhan I, Diederich K, Kaderali L, Domingues F, Albrecht M, Lohmann V, Erfle H, Bartenschlager R (2015). Identification of HNRNPK as regulator of hepatitis C virus particle production. PLoS Pathog.

[43] Hernaez B, Escribano JM, Alonso C (2008). African swine fever virus protein p30 interaction with heterogeneous nuclear ribonucleoprotein K (hnRNP-K) during infection. FEBS Lett.

[44] Netherton C, Wileman T (2013). African swine fever virus organelle rearrangements. Virus Res.

[45] Andrés G (2017). African swine fever virus gets undressed: new insights on the entry pathway. J Virol.

[46] Daecke JO, Fackler T, Dittmar MT, Krausslich HG (2005). Involvement of clathrin-mediated endocytosis in human immunodeficiency virus type 1 entry. J Virol.

[47] Naranatt P, Krishnan H, Smith M, Chandran B (2005). Kaposis sarcoma associated herpesvirus modulated microtubule dynamics via RhoA-GTP-diaphanous 2 signaling and utilizes the dynein motors to deliver its DNA to the nucleus. J Virol.

[48] Johnson C, Tinti M, Wood NT, Campbell DG, Toth R, Dubois F, Geraghty K, Wong BH, Brown LJ, Tyler J, Gernez A, Chen S, Synowsky S, MacKintosh C (2011). Visualization and biochemical analyses of the emerging mammalian 14-3-3-phosphoproteome. Mol Cell Proteomics.

[49] Ohman T, Söderholm S, Hintsanen P, Välimäki E, Lietzén N, MacKintosh C, Aittokallio T, Matikainen S, Nyman T (2014). Phosphoproteomics combined with quantitative 14-3-3-affinity capture identifies SIRT1 and RAI as novel regulators of cytosolic double-stranded RNA recognition pathway. Mol Cell Proteomics.

[50] Ohman T, Lietzen N, Valimaki E, Melchiorsen J, Matikainen S, Nyman T (2010). Cytosolic RNA recognition pathway activates 14-3-3 protein mediated signaling and caspase dependent disruption of cytokeratin network in human keratinocytes. J Proteome Res.

[51] Pei Z, Harrison M, Schmitt A (2011). Parainfluenza virus 5 m protein interaction with host protein 14-3-3 negatively affects virus particle formation. J Virol.

[52] Salas ML, Andrés G (2013). African swine fever virus morphogenesis. Virus Res.

[53] Xing H, Zhang S, Weinheimer C, Kovacs A (2000). 14-3-3 proteins block apoptosis and differentially regulate MAPK cascades. EMBO J.

[54] Samuel T, Weber H, Rauch P, Verdoodt B, Eppel JT, McShea A, Hermeking H, Funk JO (2001). The G2/M regulator 14-3-3sigma prevents apoptosis through sequestration of Bax. J Biol Chem.

[55] Cuddihy AR, O’Connell MJ (2003). Cell-cycle responses to DNA damage in G2. Int Rev Cytol.

[56] Mendes M, Pérez-Hernandez D, Vázquez J, Coelho A, Cunhaa C (2013). Proteomic changes in HEK-293 cells induced by hepatitis delta virus replication. J Proteomics.

[57] Tulman E, Delhon G, Ku B, Rock D (2009). African swine fever virus. Curr Top Microbiol Immunol.

[58] Granja AG, Nogal ML, Hurtado C, Salas J, Salas ML, Carrascosa AL, Revilla Y (2004). Modulation of p53 cellular function and cell death by African swine fever virus. J Virol.

[59] Chan YK, Gack MU (2016). A phosphomimetic-based mechanism of dengue virus to antagonize innate immunity. Nat Immunol.

[60] Argilaguet JM, Pérez-Martín E, Nofrarías M, Gallardo C, Accensi F, Lacasta A, Mora M, Ballester M, Galindo-Cardiel I, López-Soria S, Escribano JM, Reche PA, Rodríguez F (2012). DNA vaccination partially protects against African swine fever virus lethal challenge in the absence of antibodies. PLoS One.

[61] Kulichkova VA, Tsimokha AS, Fedorova OA, Moiseeva TN, Bottril A, Lezina L, Gauze LN, Konstantinova IM, Mittenberg AG, Barlev NA (2010). 26S proteasome exhibits endoribonuclease activity controlled by extra-cellular stimuli. Cell Cycle.

[62] Chen Q, Ross AC (2005). Vitamin A and immune function: retinoic acid modulates population dynamics in antigen receptor and CD38-stimulated splenic B cells. Proc Natl Acad Sci U S A.

[63] Pai T, Chen Q, Zhang Y, Zolfaghari R, Ross AC (2007). Galacto mutarotase and other galactose-related genes are rapidly induced by retinoic acid in human myeloid cells. Biochemistry.

[64] Galindo I, Hernaez B, Muñoz-Moreno R, Cuesta-Geijo MA, Dalmau-Mena I, Alonso C (2012). The ATF6 branch of unfolded protein response and apoptosis are activated to promote African swine fever virus infection. Cell Death Dis.

[65] Fung T, Huang M, Liu DX (2014). Coronavirus-induced ER stress response and its involvement in regulation of coronavirus–host interactions. Virus Res.

[66] Gomez-Gutierrez J, Elpek KG, Montes de Oca-Luna R, Shirwan H, Zhou S, McMasters K (2007). Vaccination with an adenoviral vector expressing calreticulin-human papillomavirus 16 E7 fusion protein eradicates E7 expressing established tumors in mice. Cancer Immunol Immunother.

[67] Aboud L, Ball TB, Tjernlund A, Burgener A (2014). The role of serpin and cystatin antiproteases in mucosal innate immunity and their defense against HIV. Am J Reprod Immunol.

[68] Classen CF, Bird I, Debatin KM (2006). Modulation of the granzyme B inhibitor proteinase inhibitor 9 (PI-9) by activation of lymphocytes and monocytes in vitro and by Epstein–Barr virus and bacterial infection. Clin Exp Immunol.

[69] Barrie MB, Stout HW, Abougergi MS, Miller BC, Thiele DL (2004). Antiviral cytokines induce hepatic expression of the granzyme B inhibitors, proteinase inhibitor 9 and serine proteinase inhibitor 6. J Immunol.

[70] Phillips T, Opferman JT, Shah R, Liu N, Froelich CJ, Ashton-Rickardt PG (2004). A role for the granzyme B inhibitor serine protease inhibitor 6 in CD8^+^ memory cell homeostasis. J Immunol.

